# Sciatica—Iodide of Potassium

**Published:** 1844-07

**Authors:** 


*Sciatica—Iodide of Potassium.*—Dr. Hunt, who has written a work, highly commended in the London journals, on Neuralgia, relates the following interesting case:

"A gentleman of middle age, of strong robust health, a great fisherman, was seized with a violent attack of sciatica in both legs, after having stood for many hours in the water. The violence of the attack was subdued by medical treatment; it then gradually dwindled into a chronic form, and resisted the efforts of numerous physicians and surgeons for the space of four years. The consequence of this protracted suffering was impaired health, loss of flesh, loss of spirits, of energy, and power over the lower extremities, which were wasted in a greater degree than his emaciated body. The pain was constant, with great nocturnal exacerbations, and with some degree of nightly fever. In this miserable state, after every remedy, rational and empirical, both here and in Germany, had been tried and failed, he was advised to take three grains of the iodide of potassium, in the decoction of sarsaparilla, three times a day. On the fourth night the pain was sensibly diminished; on the fifth more so; and at the expiration of ten days he was perfectly free and easy. He continued the medicine six weeks, at the end of which time he was restored to his former health; he had also recovered the use of his legs, which were ultimately restored to their usual size and firmness."

